# An Artificial Intelligence–Driven Digital Health Solution to Support Clinical Management of Patients With Long COVID-19: Protocol for a Prospective Multicenter Observational Study

**DOI:** 10.2196/37704

**Published:** 2022-10-14

**Authors:** Aïna Fuster-Casanovas, Luis Fernandez-Luque, Francisco J Nuñez-Benjumea, Alberto Moreno Conde, Luis G Luque-Romero, Ioannis Bilionis, Cristina Rubio Escudero, Irene Alice Chicchi Giglioli, Josep Vidal-Alaball

**Affiliations:** 1 Unitat de Suport a la Recerca a la Catalunya Central Gerència Territorial de la Catalunya Central Institut Català de la Salut Sant Fruitós de Bages Spain; 2 Adhera Health Inc Palo Alto, CA United States; 3 Virgen Macarena University Hospital Andalusian Health Service Sevilla Spain; 4 Research Unit Aljarafe-Sevilla Norte Health District Andalusian Health Service Sevilla Spain; 5 Department of Computer Languages and Systems University of Sevilla Sevilla Spain; 6 Health Promotion in Rural Areas Research Group Gerència Territorial de la Catalunya Central Institut Català de la Salut Sant Fruitós de Bages Spain; 7 Faculty of Medicine University of Vic-Central University of Catalonia Vic Spain

**Keywords:** COVID-19 syndrome, artificial intelligence, AI, primary health care, Postacute COVID-19 syndrome, COVID-19, health system, health care, health care resource, public health policy, long COVID-19, mHealth, digital health solution, patient, clinical information, clinical decision support

## Abstract

**Background:**

COVID-19 pandemic has revealed the weaknesses of most health systems around the world, collapsing them and depleting their available health care resources. Fortunately, the development and enforcement of specific public health policies, such as vaccination, mask wearing, and social distancing, among others, has reduced the prevalence and complications associated with COVID-19 in its acute phase. However, the aftermath of the global pandemic has called for an efficient approach to manage patients with long COVID-19. This is a great opportunity to leverage on innovative digital health solutions to provide exhausted health care systems with the most cost-effective and efficient tools available to support the clinical management of this population. In this context, the SENSING-AI project is focused on the research toward the implementation of an artificial intelligence–driven digital health solution that supports both the adaptive self-management of people living with long COVID-19 and the health care staff in charge of the management and follow-up of this population.

**Objective:**

The objective of this protocol is the prospective collection of psychometric and biometric data from 10 patients for training algorithms and prediction models to complement the SENSING-AI cohort.

**Methods:**

Publicly available health and lifestyle data registries will be consulted and complemented with a retrospective cohort of anonymized data collected from clinical information of patients diagnosed with long COVID-19. Furthermore, a prospective patient-generated data set will be captured using wearable devices and validated patient-reported outcomes questionnaires to complement the retrospective cohort. Finally, the ‘Findability, Accessibility, Interoperability, and Reuse’ guiding principles for scientific data management and stewardship will be applied to the resulting data set to encourage the continuous process of discovery, evaluation, and reuse of information for the research community at large.

**Results:**

The SENSING-AI cohort is expected to be completed during 2022. It is expected that sufficient data will be obtained to generate artificial intelligence models based on behavior change and mental well-being techniques to improve patients’ self-management, while providing useful and timely clinical decision support services to health care professionals based on risk stratification models and early detection of exacerbations.

**Conclusions:**

SENSING-AI focuses on obtaining high-quality data of patients with long COVID-19 during their daily life. Supporting these patients is of paramount importance in the current pandemic situation, including supporting their health care professionals in a cost-effective and efficient management of long COVID-19.

**Trial Registration:**

Clinicaltrials.gov NCT05204615; https://clinicaltrials.gov/ct2/show/NCT05204615

**International Registered Report Identifier (IRRID):**

DERR1-10.2196/37704

## Introduction

### Definition of Long COVID-19

A percentage of people report prolonged and recurrent symptoms, for weeks or months, after the first episode of COVID-19. Persistent COVID-19 has not yet been precisely defined. It seems clear that it is a disease that affects a large number of people, generating a huge health and social impact in the pandemic [1]. In this context, patient groups, the international scientific community, as well as public health institutions and authorities are making a great effort to improve knowledge, clinical care, and social benefits [2,3]. Numerous Spanish scientific societies alongside patient groups have drawn up a Clinical Guide in which long COVID-19 is defined [2] as follows:

“Multiorgan symptomatic complex that affects those patients who have suffered from COVID-19 (with or without diagnosis confirmed by laboratory tests) and who remain with symptomatology once passed the considered acute phase of the disease, after 4 or even 12 weeks, with symptoms persisting over time.”

### Incidence of Long COVID-19 and the Most Affected Population

Determining the incidence of long COVID-19 is complicated due to the absence of specific surveillance and a variety of definitions. Another difficulty is that studies are performed in selected groups of patients, which does not allow estimating the true incidence in the population. The UK National Institute of Statistics estimated that 1 in 5 people with COVID-19 had symptoms beyond 5 weeks, and 1 in 10 people had symptoms beyond 12 weeks [4]. In a population-based survey in the United States, the percentage of people with persistent symptoms was 30% at 30 days, 25% at 60 days, and 15% at 90 days [5].

In the UK National Institute of Statistics study [6], a higher incidence was observed in women (23.6%, 95% CI 22.2-25.0) versus men (20.7%, 95% CI 19.3-22.1) and in the middle age. In the US survey, the only factor associated with persistent COVID-19, measured by the number of symptoms, was the initial severity [7].

### Long Covid-19 Fatigue and Beyond: Pathophysiology, Symptoms, and Signs

The pathophysiological basis remains unknown, and several theories are put forward: the persistence of the virus in reservoirs, such as the small intestinal epithelium, where it would remain active [8,9]; the presence of an aberrant immune response [10]; the damage produced by the effect of autoantibodies against immunomodulatory proteins [11]; or the hyperactivation of coagulation and platelets [12]. The symptoms and signs are extremely numerous and varied (ie, systemic, neurological, psychiatric, and cardiovascular) [13-19].

It is important to know the people who experience long COVID-19 for a better characterization [20], since it is a characteristically intermittent disease with very varied symptoms, which also involves people who at the time did not have access to a diagnostic test and those affected who felt stigmatized and ignored [21].

A web-based survey by the Spanish Society of General and Family Physicians in 1834 participants reported a variety of more than 200 symptoms. These symptoms included fatigue and general malaise in more than 95% of the patients. Headache, low mood, and muscle aches were observed in more than 80% of them. Dyspnea, joint, chest, and back pain, as well as lack of concentration were detected in more than 75% of them. More than 70% found it difficult to attend their daily duties, and more than 30% reported difficulties even with personal hygiene. Although 52% of the cases were not confirmed by laboratory diagnostic tests, the authors noted that there were no significant differences between the groups with or without diagnostic confirmations [19].

### Background and Current Status

COVID-19 caused serious problems to the health system, collapsing it, and depleting the health resources available [20-22]. Fortunately, the development and enforcement of specific public health policies, such as vaccination, mask wearing, and social distancing, among others, has reduced the prevalence and complications associated with COVID-19 in the acute phase [23]. However, the aftermath of the global pandemic requires an efficient approach to manage patients with long COVID-19. It seems a great opportunity to leverage on innovative digital health solutions to provide exhausted health care systems with the most cost-effective and efficient tools available to support the clinical management of the population experiencing long COVID-19 [24,25].

We can find more than 250 health-labeled apps available on both Google Play and Apple Store, but they are basic products that offer neither the technology nor the advanced approach and services offered to patients with long COVID-19. Adhera Health Fatigue Digital Program for long COVID-19 is built on the principles of patient centricity, and it is guided by the principles of participatory research to promote a meaningful partnership between patients and health care professionals [26]. Furthermore, data mining and artificial intelligence (AI) have been greatly applied lately to health care areas [27]. Data mining is a combination of statistical analysis, algorithms, AI, and database management, with the purpose of extracting intelligence. AI can be defined as the field devoted to build artificial creatures. It is the science and engineering of making intelligent machines, especially intelligent computer programs. In the last years, there has been a growing interest in the application of AI and data mining techniques to clinical data. MEDLINE has seen a sharp 10-fold increase in the number of papers having the term ‘data mining’ in their title [28]. This AI-driven research is designed toward the improvement of the Adhera Precision Digital Companion platform toward the provision of advanced personalization technologies for adaptive self-management, which is a progress beyond the state of the art [29,30].

The objective of this protocol is the prospective collection of psychometric and biometric data for training algorithms and prediction models to complement the SENSING-AI cohort. Likewise, the final aim of the project is the creation of a digital health solution based on AI and prediction models for a better clinical management of patients with long COVID-19 and to improve self-management of this condition.

## Methods

### Ethics Approval

This study was approved by the research ethics committee of Primary Care Research Institute Jordi Gol (in Barcelona, Spain) (Código CEIm: 22/010-PCV) and Virgen Macarena University Hospital (Seville, Spain) (1894-N-21). All patients will receive a patient information sheet and will sign an informed consent.

### Participation Consent and Protection of Personal Data

This study is registered in Clinicaltrials.gov website (NCT05204615). Data obtained from patients will be pseudo-anonymized by the clinical partner. Only a code, based on an alphanumeric number completely unlinked to any direct patient data, will be included as an identifier of the subjects in the study.

#### Design

This is a prospective multicenter observational study to complement the SENSING-AI cohort.

#### Sample Size

Considering that there is no previous experience with long COVID-19, the sample size for the prospective data collection is a small cohort of patients (N=10) to assess the quality of the data and the feasibility of the study. Based on the results obtained, we plan to expand the cohort of patients. In this context, 10 patients with long COVID-19 will be recruited and followed up for 4 weeks at designated primary care centers. Of them, 5 patients will be recruited by the team of the Aljarafe-Seville North Health District of Andalusian Public Foundation for Health Research Management of Seville (FISEVI) and the other 5 by the team of the Primary Care Research Institute (IDIAP) Jordi Gol. The inclusion criteria for participants will be as follows: (1) patients over the age of 18 years; (2) patients diagnosed with persistent COVID-19 in the past year; and (3) having symptoms of fatigue, dyspnea, shortness of breath, anxiety, stress, depression, conduct disorder, or sleep disorder.  The exclusion criteria will be as follows: (1) hospital admission during follow-up period motivated by pathology and not related to COVID-19; (2) patients without technological knowledge or unable to use the mobile app; (3) having a known severe psychiatric illness or cognitive impairment; (4) pregnant women; or (5) patients discharged after hospital admission due to COVID-19. 

#### Procedures

AI models will be generated from the following 3 data sources: (1) review of publicly available data sources (eg, OpenAIRE, FAIRsharing, National Sleep Research Resource, DEAP data set, and Kaggle) related to long COVID-19; (2) cohort of anonymized retrospective data (ie, 100 cases) obtained from clinical information from patients with COVID-19, attended by the primary care teams of the Seville North health district; and (3) prospective data collected using Adhera Health Digital Precision Companion platform, which includes clinical, biometric, and psychometric data from 10 patients followed during 1 month by FISEVI and IDIAP Jordi Gol.

#### Data Collection

Wearable devices will be used to collect data in real time for 1 month to detect physiological and psychological complications.

Biometric information will be collected from wearable devices (Withings Scanwatch) provided to patients. The data to be collected from each patient are classified in Table 1.

The Adhera Health’s sensing module will allow the collection of psychometric data using mobile-based validated questionnaires and the integration of wearable data. Based on previous literature [13-19], the most relevant psychometric data for the generation of prediction models were considered to be related to fatigue, dyspnea, anxiety, stress, depression, and sleep disorder. The data to be obtained is classified in Table 2 [31-36].

**Table 1:** Data collected by wearable devices.

Biometric data	Types of data
Activity data	Daily distance traveled in meters Daily number of steps: list of steps per 4-5 minutes approximately Total daily calories burned in kcal
Training data	Calories or event in kcal Distance of the event in meters Heart rate; minimum, average, and maximum intensity in beats per minute
Sleep data	Ratio of total sleep time or time spent in bed Time spent awake in bed after falling asleep for the first time during the night in seconds REM^a^ sleep phase count
Cardiac data	Atrial fibrillation detected in seconds during an ECG^b^ Detailed ECG signal in μV with 300 Hz sampling rate

^a^REM: rapid eye movement.

^b^ECG: electrocardiogram.

**Table 2:** Data collected by validated questionnaires using Adhera Precision Digital Companion platform.

Psychometric data	Type of data
Fatigue	Weekly FAS^a^ questionnaire
Dyspnea	Weekly MEG DI^b^ questionnaire
Anxiety	Weekly GAD^c^ questionnaire
Stress	Weekly PSS-10^d^ questionnaire
Depression	Weekly PHQ^e^ questionnaire
Sleep disorder	Weekly questionnaire

^a^Fatige assessment scale.

^b^Melbourne ENT group dyspnea index.

^c^General anxiety disorder.

^d^Perceived stress scale.

^e^Patient health questionnaire.

### Analysis of the Cohort Data

A prediction algorithm based on the nearest neighbor classification method will be used. This method is an instance-based algorithm supervised by machine learning. These algorithms will process the data flows in which the input is presented as a sequence of elements. Therefore, it will allow for searching in the closest observations. This algorithm cannot provide human interpretable models; processing procedures will be applied to make them explainable, based on feature classification. Therefore, this model has the aim of predicting whether the user is having a complication, based on clinical, biometric, and psychometric data.

By the data obtained in the prospective study, an adaptive adjustment of the sampling frequency of the ecological momentary assessments will be made. The objective is to develop a model to predict the most appropriate time to activate the validated questionnaire for the patient. This model will be developed using machine learning algorithms, mainly based on decision trees. These are flowchart-like structures in which each node represents a value in an entity, each branch represents the value, and each leaf represents a class or decision label after calculating all attributes. The model will be measured based on error rates and confusion tables, which will allow measuring accuracy, precision, F1 score, sensitivity, specificity, receiver operating characteristic curve, and area below the hamstring curve.

Another model will be developed to adapt the questionnaires to each patient. It will be focused on the user’s history and biometric measurements; the model will decide the number of questions needed for that patient. To develop this model, machine Learning models will be trained, mainly based on artificial neural networks. These are based on a collection of connected units or nodes called artificial neurons, which freely model the neurons in a biological brain. An artificial neuron receives a signal, processes it, and then signals to neurons connected to it. For each question, the input data will be the entities, and the output data will be a predicted answer to the question. If the question is easily predicted, the question will be removed from the test. If not, it has to remain in the test and be answered by the patient. This type of algorithm is not human interpretable, and therefore, postprocessing models will be applied to make them explainable.

## Results

The study is registered in clinical trials, and the SENSING-AI cohort is expected to be completed during 2022.

It is expected that sufficient data will be obtained to generate AI models to enhance the AI-precision digital companion solution toward the provision of adaptive self-management in patients with long COVID-19, while providing useful and timely clinical decision support services to health care professionals based on risk stratification models and early detection of exacerbations.

## Discussion

The development of an AI-driven digital health solution based on behavior change techniques will help improve the clinical management of patients with long COVID-19 and improve their well-being and quality of life.

This research focuses on maximizing the usefulness of the information that can be generated by the patient using AI techniques. Once COVID-19 has been controlled in the acute phase through vaccination, it is time to generate new resources focused on long COVID-19. In this context, it is necessary to develop solutions for the detection of exacerbating disease at an early stage, improving patient care, and improving clinical prognosis. It is also necessary to provide AI tools, incorporating monitors to obtain automatic, objective, and easy-to-interpret data for professionals. These tools will be of considerable benefit to professionals, as they will be able to obtain a risk stratification of disease complications in real time, increasing the capacity for case management and aiding in critical decisions.

Technological progress in recent decades has had a great impact on the volume of information. The management of research data is implemented continuously throughout its life cycle. It starts at the planning stage, and it continues with the execution and dissemination of results and the preservation of data. Achieving good data management allows for generating greater innovation and knowledge. In this context, the 4 principles called FAIR arise, oriented to favor maximum performance of the data obtained in research. Applying these principles has significant benefits for the scientific community, improving the flow of information, maximizing the performance of the data obtained, and promoting the improvement of research in patients with long COVID-19. To promote research and reuse of data in future studies, the intention of this project is to make the data FAIR. The data obtained in the study will be discoverable, accessible, interoperable, and reusable.

This study has several limitations. First, the study is focused on a localized population. Even so, one part is representative of a rural population and the other is more urban. Being an innovative study without previous data, once the data are all collected, it is possible that some necessary variables have not been noticed. Likewise, making the data FAIR will help future research to improve this possible limitation. Finally, there may be a sample bias, since the population recruited are adults with different levels of digital skills.

## Abbreviations

FAIR: Findability, Accessibility, Interoperability, and Reuse
FISEVI: Fundación Pública Andaluza para la Gestión en la Investigación en Salud de Sevilla (Andalusian Public Foundation for Health Research Management of Seville)
IDIAP: Primary Health Care Research Institute
